# ABL2 suppresses FLT3-ITD-induced cell proliferation through negative regulation of AKT signaling

**DOI:** 10.18632/oncotarget.14577

**Published:** 2017-01-10

**Authors:** Julhash U. Kazi, Kaja Rupar, Alissa Marhäll, Sausan A. Moharram, Fatima Khanum, Kinjal Shah, Mohiuddin Gazi, Sachin Raj M. Nagaraj, Jianmin Sun, Rohit A. Chougule, Lars Rönnstrand

**Affiliations:** ^1^ Division of Translational Cancer Research, Department of Laboratory Medicine, Lund University, Lund, Sweden; ^2^ Lund Stem Cell Center, Department of Laboratory Medicine, Lund University, Lund, Sweden; ^3^ Department of Pathogen Biology and Immunology, School of Basic Medical Sciences, Ningxia Medical University, Yinchuan, P. R. China; ^4^ Division of Oncology, Skåne University Hospital, Lund, Sweden

**Keywords:** ABL2, ARG, FLT3-ITD, AKT, AML

## Abstract

The type III receptor tyrosine kinase FLT3 is one of the most commonly mutated oncogenes in acute myeloid leukemia (AML). Inhibition of mutated FLT3 in combination with chemotherapy has displayed promising results in clinical trials. However, one of the major obstacles in targeting FLT3 is the development of resistant disease due to secondary mutations in FLT3 that lead to relapse. FLT3 and its oncogenic mutants signal through associating proteins that activate downstream signaling. Thus, targeting proteins that interact with FLT3 and their downstream signaling cascades can be an alternative approach to treat FLT3-dependent AML. We used an SH2 domain array screen to identify novel FLT3 interacting proteins and identified ABL2 as a potent interacting partner of FLT3. To understand the role of ABL2 in FLT3-mediated biological and cellular events, we used the murine pro-B cell line Ba/F3 as a model system. Overexpression of ABL2 in Ba/F3 cells expressing an oncogenic mutant of FLT3 (FLT3-ITD) resulted in partial inhibition of FLT3-ITD-dependent cell proliferation and colony formation. ABL2 expression did not alter the kinase activity of FLT3, its ubiquitination or its stability. However, it partially blocked FLT3-induced AKT phosphorylation without affecting ERK1/2 and p38 activation. Taken together our data suggest that ABL2 acts as negative regulator of signaling downstream of FLT3.

## INTRODUCTION

The mammalian genome encodes more than 500 protein kinases that contribute to the regulation of almost all cellular events. Around 60 protein kinases are characterized as receptor tyrosine kinases which are regulated by extracellular stimuli including growth factors [1, 2]. The type III receptor tyrosine kinase family includes the receptors for platelet derived growth factors (PDGFRA and PDGFRB), the receptor for stem cell factor (SCFR or KIT), FMS-like tyrosine kinase 3 (FLT3, the receptor for FLT3 ligand, FL) and the colony-stimulating factor 1 receptor (CSF1R). Several members of this family are important regulators of the hematopoietic system and have been implicated in various hematological malignancies including acute myeloid leukemia (AML). AML originates from the myeloid lineage of hematopoietic cells [3] and more than 30% of AML patients carry an oncogenic mutation in the FLT3 gene [4]. FLT3 and other type III receptor tyrosine kinases share common domain arrangements such as an extracellular ligand binding domain, a transmembrane domain, a juxtamembrane domain and a kinase domain (split by a short kinase insert). Its ligand, FL, forms spontaneous dimers and binds to the extracellular domain of FLT3 and thereby induces dimerization of FLT3 which further promotes activation of its intrinsic kinase activity and autophosphorylation on several tyrosine residues. Phosphotyrosine residues are well-known as docking sites for SH2 domain-containing signaling proteins that regulate, depending on the characteristic of the partner protein, either the activation or inhibition of signaling downstream of the receptor. For instance, ubiquitin E3 ligases such as CBL, SOCS2 and SOCS6 bind to FLT3 and negatively regulate downstream signaling. In contrast, the adaptor proteins GRB10 [5] and GADS [6], and the non-receptor tyrosine kinases SYK [7] and FYN [8], enhance downstream signaling.

The mammalian Abelson (ABL) family of non-receptor tyrosine kinase includes the two members ABL and ABL2 (also called ARG, ABL-related gene). ABL and ABL2 transduce signals from upstream receptors and regulate numerous biological processes such as cell survival, apoptosis, response to genotoxic stress, morphogenesis and cell motility [9]. ABL family kinases have been implicated in leukemia as the BCR-ABL fusion gene [10]. The BCR-ABL fusion gene is the major oncogene in chronic myelogenous leukemia (CML). The BCR-ABL fusion gene has also been reported less frequently in acute lymphoblastic leukemia (ALL) and rarely in AML [11]. Like ABL, ABL2 forms fusion genes with TEL transcription factors. However, this is a rare event in AML [12]. Recent studies suggest that, besides gene fusions, expression of ABL family kinases is upregulated in several cancers such as pancreatic cancer, anaplastic thyroid cancers, colorectal cancer, melanoma and non-small-cell lung cancers [13–16]. ABL family kinases regulate invasion, proliferation and survival mediated by the epidermal growth factor receptor (EGFR), the insulin-like growth factor receptor (IGFR), HER2 and SRC kinases [17–21]. However, the role of this family of kinases has not been studied with respect to signaling downstream of FLT3. Here we identify ABL2 as an FLT3 interacting protein and show that ABL2 plays a role in signaling downstream of FLT3.

## RESULTS

### Identification of ABL2 as a FLT3 binding protein

**Figure 1:**
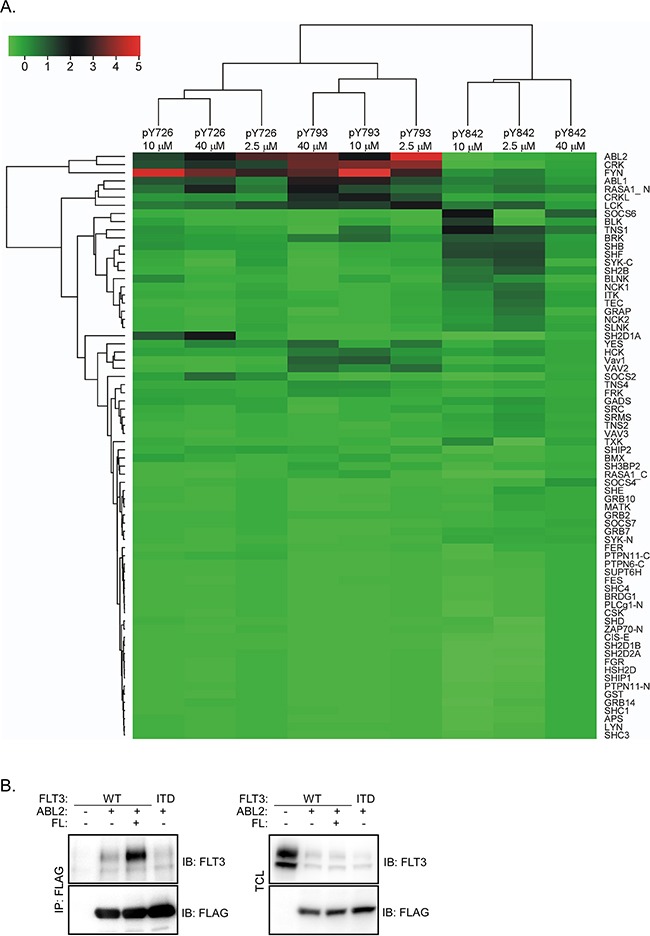
ABL2 binds to FLT3 in response to FL stimulation **A**. An SH2 domain array screen identifies ABL2 as a novel FLT3-associating protein. Seventy-four SH2 domains from different proteins were used. **B**. COS-1 cells were transfected with plasmids expressing FLT3-WT and empty vector or FLAG-tagged ABL2. After 24 hours of transfection cells were stimulated for 5 minutes with 100 ng/ml FL followed by lysis. Lysates were immunoprecipitated using an anti-FLAG antibody.

Receptor tyrosine kinases such as FLT3 signal through proteins that associate with the activated receptor. In order to identify novel FLT3-interacting proteins we used an SH2 domain array [22]. Seventy-four recombinant SH2 domains from 64 different proteins were used. Three different tyrosine phosphorylated peptides corresponding to residues Y726, Y793 and Y842 in FLT3 were used to determine the binding to the SH2 domains. We observed that the ABL2 SH2 domain displayed the highest affinity to the tyrosine phosphorylated FLT3 peptides (Figure 1A). Other associating proteins included several SRC family kinases, SOCS6, ABL, CRK, CRKL etc. Furthermore, we could show that association between ABL2 and FLT3 is FL-dependent and that oncogenic FLT3-ITD displays constitutive association with ABL2 (Figure 1B).

### ABL2 expression negatively regulates FLT3-ITD-mediated cell viability and colony formation

**Figure 2:**
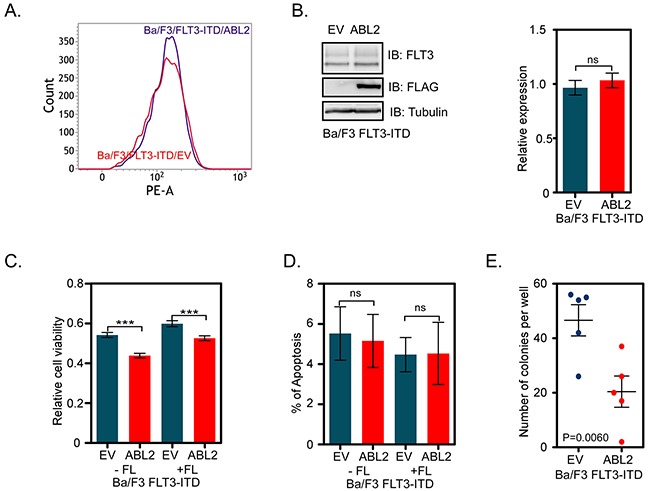
ABL2 expression reduces FLT3-ITD-mediated cell viability and colony formation **A**. Ba/F3-FLT3-ITD cells expressing ABL2, or empty vector were labeled with phycoerythrin-conjugated anti-FLT3 antibody. Samples were then analyzed by flow cytometry. **B**. Ba/F3-FLT3-ITD cells expressing ABL2, or empty vector were lysed. Equal amount of proteins from total cell lysates were subjected to Western blot analysis. Blots from multiple experiments were quantified and analyzed using student's t-test. **C**. Ba/F3 cells expressing FLT3-ITD and ABL2 or FLT3-ITD and empty vector were used for the PrestoBlue cell viability assay. **D**. Cells positive for Annexin V and 7-aminoactinomycin D (7-AAD) or only for Annexin V were counted as apoptotic cells. **E**. Cells were washed to remove IL-3 and serum. Cells were mixed with 80% methylcellulose medium and seeded in a 24-well plate. ns, not significant; ***, p<0.001.

To explore the role of ABL2 in oncogenic FLT3-ITD-mediated biological responses, we generated Ba/F3 cells overexpressing both FLT-ITD and ABL2 or empty control vector (EV). FLT3-ITD surface expression was analyzed by flow cytometry and the total expression was determined by western blotting. The results demonstrate an equal surface (Figure 2A) as well as total (Figure 2B) expression of FLT3-ITD in both ABL2 and EV expressing cells. We used these cell lines to determine the dependency of ABL2 for cell viability and colony formation. Cell viability was determined using the dye PrestoBlue. We observed that cells expressing ABL2 displayed significantly reduced viability when compared to cells expressing empty control vector (Figure 2C). However, ABL2 expression did not affect the apoptotic rate (Figure 2D). The potential to form colonies in semi-solid medium was also reduced in cells expressing ABL2 (Figure 2E).

### ABL2 expression negatively regulates AKT phosphorylation

**Figure 3:**
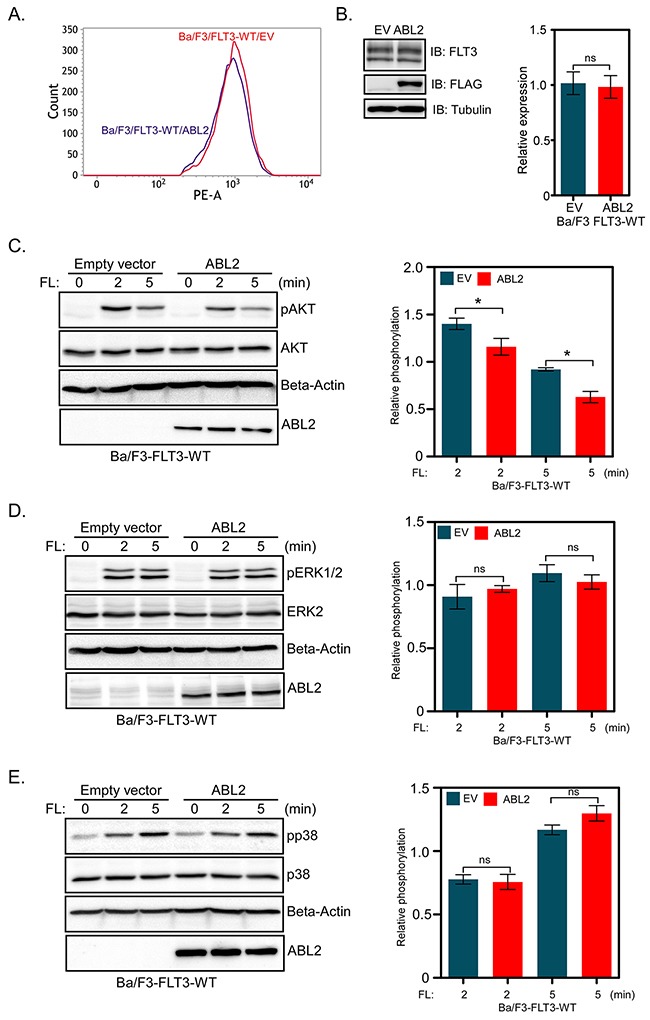
Ba/F3-FLT3 cells expressing ABL2 display reduced AKT phosphorylation **A**. Ba/F3 cells expressing FLT3 and ABL2 or empty vector were labeled with phycoerythrin-conjugated anti-FLT3 antibody and then analyzed by flow cytometry. **B**. Ba/F3-FLT3-ABL2 and Ba/F3-FLT3-EV cells were lysed, and lysates were analyzed by Western blotting. Blots from multiple experiments were quantified and analyzed using student t-test. (C-E) Ba/F3-FLT3-WT cells expressing ABL2 or empty vector were washed to remove IL-3 and serum starved four hours before FL stimulation. Total cell lysates were used for Western blotting analysis using anti-phospho-AKT antibody **C**., anti-phospho-ERK1/2 antibody **D**. and anti-phospho-p38 antibody **E**. Multiple blots were quantified and subjected to statistical analysis. ns, not significant; *, p<0.05.

Wild-type FLT3 activates the PI3K/AKT, RAS/ERK and p38 pathways in response to FL-stimulation while FLT3-ITD constitutively activates those pathways [23–25]. Since signaling downstream of the wild-type receptor is controlled by FL stimulation, we generated Ba/F3 cell lines expressing wild-type FLT3 and ABL2 or EV in order to study how ABL2 regulates signaling downstream of FLT3. Flow cytometry and western blotting experiments verified equal surface (Figure 3A) as well as total (Figure 3B) expression of wild-type FLT3 in cells expressing ABL2 or empty vector. The expression of ABL2 partially blocked FLT3-mediated AKT phosphorylation (Figure 3C). However, ABL2 expression did not alter FL-induced ERK1/2 (Figure 3D) or p38 (Figure 3E) phosphorylation.

### ABL2 expression has no effect on FLT3 activation, ubiquitination or degradation

**Figure 4:**
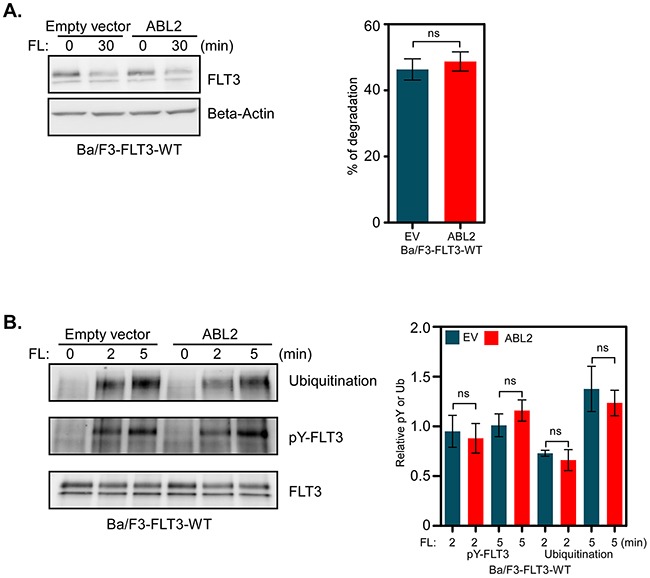
ABL2 expression does not alter FLT3 activation, ubiquitination or degradation **A**. Cells were incubated with cycloheximide before stimulation with FL. Total cell lysates were used for Western blot analysis. Multiple blots were quantified for statistical analysis. **B**. Ba/F3-FLT3-WT cells expressing ABL2 or empty vector were washed to remove IL-3 and serum starved for four hours followed by FL stimulation, lysis and immunoprecipitation using an anti-FLT3 antibody.

A majority of the proteins that associate with receptor tyrosine kinases affect either receptor activation, ubiquitination or stability [26, 27]. We analyzed whether ABL2 has a role in regulating FLT3 protein stability. Ba/F3-FLT3-EV and Ba/F3-FLT3-ABL2 cells were treated with cycloheximide for 30 minutes, in order to block protein synthesis, and then stimulated with FL for 30 minutes in the presence of cycloheximide. We did not observe any difference in FLT3 degradation whether ABL2 was overexpressed or not (Figure 4A). ABL2 expression neither altered FL-induced tyrosine phosphorylation nor ubiquitination of FLT3 (Figure 4B).

ns, not significant.

## DISCUSSION

Signaling downstream of receptor tyrosine kinases is tightly controlled by various associating proteins such as kinases, phosphatases, ubiquitin E3 ligases, adaptors and scaffolding proteins. In order to maintain a proper balance in signaling, many interacting proteins enhance receptor signaling, but some of them turn off the signal by dephosphorylating the receptor or directing the receptor for ubiquitin-mediated degradation. Thus, the level of signaling is maintained at a level that is appropriate for the cell. Too much RTK signaling can be deleterious and lead to induction of apoptosis [28]. Here we identify ABL2 as a novel FLT3-associating protein using an SH2 domain array screening. We show that ABL2 negatively regulates FLT3-ITD-mediated biological events through partial inhibition of AKT signaling.

The recombinant SH2 domain of ABL2 displayed a high affinity for multiple phosphopeptides corresponding to known FLT3 tyrosine phosphorylation sites. In addition to the ABL2 SH2 domain, the SH2 domains of CRK, CRKL, FYN, ABL, LCK, SOCS6, SYK etc. also displayed a considerable affinity for those phosphopeptides. Many of those proteins have already been verified by biochemical methods and identified as critical regulators of FLT3 signaling [7, 8, 29]. ABL2 associates with FLT3 only when FLT3 is activated, suggesting that FLT3 tyrosine phosphorylation is required for the interaction and that the interaction is mediated through pY-SH2 binding.

FLT3 and its oncogenic counterpart, FLT3-ITD, exert survival, proliferative and transforming signals by activating mainly three different signaling cascades, i. e. the PI3K/AKT, RAS/ERK and p38 pathways [24, 25]. When ABL family proteins form fusion proteins with BCR or TEL they mediate signals that lead to cell proliferation, survival and transformation [12]. Depletion of ABL2 in non-small cell lung carcinoma cell lines led to decreased cell growth [30]. However, the effect of ABL2 expression seems to some extent to be context dependent, since in a breast cancer model, loss of ABL2 accelerated tumor growth by enhancing cell proliferation [21]. In line with these findings, we also observed that overexpression of ABL2 reduced FLT3-ITD-induced cell proliferation as well as colony formation. Although ABL2 is a tyrosine kinase, overexpression of ABL2 did not increase the total tyrosine phosphorylation of FLT3 suggesting that ABL2 is not involved in the FLT3 activation process. Furthermore, association of ABL2 did not affect FLT3 ubiquitination or degradation. Therefore, it is likely that ABL2 has no direct effect on FLT3 but that association of ABL2 with FLT3 disrupts selective downstream signaling pathways e. g. the AKT pathway. In contrast to its close relative ABL1, ABL2 seems to be a negative regulator of signaling and transformation. This observation may have different explanations: ABL2 might compete for binding to FLT3 with proteins essential for activation of the AKT pathway or ABL2 directly targets FLT3 downstream signaling proteins. The mechanism behind these signaling events will be further investigated in future studies.

## MATERIALS AND METHODS

### Reagents and antibodies

Lipofectamine 2000 and horseradish peroxidase (HRP)-coupled secondary anti-mouse and anti-rabbit antibodies were from ThermoFisher Scientific (Waltham, MA). Cycloheximide and mouse anti-FLAG (M2) antibody were from Sigma-Aldrich (St Louis, MI). FLT3 ligand (FL) was from ORF Genetics (Reykjavik, Iceland). Rabbit anti-phospho-AKT (pSer473), mouse anti-phosphotyrosine (4G10), mouse anti-mono-ubiquitin antibodies were from Abcam (Cambridge, UK), Merck Millipore (Billerica, MA) and Covance Research Products (Princeton, NJ), respectively. Rabbit anti-phospho-ERK1/2 (pThr202/pThr204), goat anti-AKT, rabbit anti-ERK2 and anti-goat secondary antibodies were from Santa Cruz Biotechnology (Santa Cruz, CA). The phycoerythrin-labeled anti-FLT3, mouse anti-phospho-p38 and anti-p38 antibodies were from BD Transduction Laboratories (Franklin Lakes, NJ). Rabbit anti-FLT3 antibody was described previously [29].

### Plasmids

Expression plasmids pcDNA3-FLT3-WT, pcDNA3-FLT3-ITD, pMSCVpuro-FLT3-WT and pMSCVpuro-FLT3-ITD plasmids were previously described [29]. Human full-length ABL2 plasmids in pCMV-Myc-FLAG vector was obtained from OriGene (Rockville, MD). Retroviral plasmid pMSCVneo-ABL2-WT-Myc-FLAG was generated by ligating ABL2-WT-Myc-FLAG fragment into the pMSCVneo vector.

### Cell culture

COS-1 and Ba/F3 cells were purchased from DSMZ (Braunschweig, Germany). EcoPack cells were from Clontech (Mountain View, CA). Dulbecco's modified Eagle's medium (DMEM) supplemented with 10% fetal bovine serum (FBS), 100 units/ml penicillin and 100μg/ml streptomycin was used for maintaining COS-1 and EcoPack cells. RPMI 1640 medium supplemented with 10% heat-inactivated FBS, 10 ng/ml recombinant murine interleukin-3 (IL-3), 100 units/ml penicillin and 100μg/ml streptomycin was used for Ba/F3 cells.

### Transient transfection

Lipofectamine 2000 was used for transient transfection. COS-1 cells were transfected with pCMV-Myc-FLAG and pcDNA3 plasmids according to the manufacturer's protocol.

### Stable transfection

Retroviral vector pMSCV was used to produce virus particles in EcoPack packaging cells. EcoPack cells were transfected with 10μg pMSCVpuro-FLT3-WT, pMSCVpuro-FLT3-ITD, pMSCVneo-ABL2-Myc-FLAG or pMSCVneo constructs using Lipofectamine 2000 followed by collection of virus-containing supernatants after 72 hours of transfection. Then Ba/F3 cells were infected with FLT3-WT or FLT3-ITD virus particles. Cells were selected using 1.2 μg/ml puromycin for two weeks and FLT3 expression was checked by flow cytometry and Western blotting. Ba/F3-FLT3-WT and Ba/F3-FLT3-ITD cells were further infected with ABL2 or empty vector virus particles and then further selected against 0.8 mg/ml G-418 for 2 weeks. ABL2 expression was verified using Western blotting. All stably transfected Ba/F3 cells were maintained in Ba/F3 medium as described above and recommended previously [31].

### Immunoprecipitation and western blotting

Ba/F3 cells were washed three times and then starved of IL3 and serum for 4 hours in RPMI-1640 before stimulation with 100 ng/ml of FL. Cells were then washed with ice-cold PBS and lysed in 1% Triton X-100 lysis buffer. For immunoprecipitation of 1 ml cell lysates one μg primary antibody was used. The Western blotting and immunodetection procedures were described elsewhere [32, 33].

### SH2 domain array screening

Screening of SH2 domain specificity of the various phosphopeptides was performed by Dr. Shawn Li's Laboratory, University of Ontario, as a service. Peptide synthesis, array constructions and assay conditions were essentially as described elsewhere [34].

### Cell viability

Ba/F3-FLT3-ITD cells expressing ABL2 or empty vector were washed three times and resuspended in RPMI 1640 supplemented with 10% FBS, 100 units/ml penicillin and 100μg/ml streptomycin. Cells were then seeded in a 96-well plate (10,000 cells per well). Two days after seeding, cell viability was counted by PrestoBlue (Molecular Probes, Eugene, OR) assay.

### Apoptosis

Ba/F3-FLT3-ITD cells expressing ABL2 or empty vector were washed three times and resuspended in RPMI 1640 supplemented with 10% FBS, 100 units/ml penicillin and 100μg/ml streptomycin. Cells were then seeded in a 12-well plate (100,000 cells per well). Two days after seeding, apoptosis was measured by flow cytometry using Annexin V and 7-aminoactinomycin D (7-AAD) apoptosis kit (BD Biosciences, Franklin Lakes, NJ).

### Colony formation assay

Ba/F3-FLT3-ITD cells expressing ABL2 or empty vector were washed three times and 1,000 cells were mixed with 500 μl of 80% methylcellulose medium and seeded in a 24-well plate. Cells were incubated for 7 days and colonies were counted by two individual researchers.

### FLT3 degradation assay

Ba/F3-FLT3-WT cells expressing ABL2 or empty vector were washed three times with PBS and treated with 100 μg/ml cycloheximide for 30 minutes. Cells were then stimulated with FL for 30 minutes in presence of cycloheximide followed by lysis in Triton X-100 lysis buffer. Cell lysates were subjected to the Western blotting analysis.

### Statistical analysis

Blots were quantified using ImageJ software and statistical analysis was performed using GraphPad Prism 5.0. Data were presented as the mean ± SE. One-way ANOVA with Bonferroni's post-tests and student's t-test were used to check statistical significance.
